# Follicular fluid from women with polycystic ovary syndrome induces granulosa cells metabolic dysfunction that is exacerbated by obesity

**DOI:** 10.3389/fendo.2026.1783780

**Published:** 2026-03-02

**Authors:** Mafalda V. Moreira, Bárbara Guerra-Carvalho, David F. Carrageta, Daniela Sousa, Raquel Brandão, Carla Leal, Emídio Vale-Fernandes, Anna Ptak, Duarte Pignatelli, Raquel L. Bernardino, Mariana P. Monteiro

**Affiliations:** 1 Unit for Multidisciplinary Research in Biomedicine (UMIB), School of Medicine and Biomedical Sciences (ICBAS), University of Porto, Porto, Portugal; 2 i3S - Instituto de Investigação e Inovação em Saúde, Universidade do Porto, Porto, Portugal; 3 LAQV-REQUIMTE, Department of Chemistry, University of Aveiro, Aveiro, Portugal; 4 ITR - Laboratory for Integrative and Translational Research in Population Health, Porto, Portugal; 5 Centre for Medically Assisted Procreation, Public Gamete Bank, Gynecology Department, Centro Materno-Infantil do Norte Dr. Albino Aroso (CMIN), Centro Hospitalar Universitário de Santo António (CHUdSA), Unidade Local de Saúde de Santo António (ULSSA), Porto, Portugal; 6 Laboratory of Physiology and Toxicology of Reproduction, Institute of Zoology and Biomedical Research, Faculty of Biology, Jagiellonian University, Krakow, Poland; 7 Department of Endocrinology, Centro Hospitalar e Universitário de S. João, Porto, Portugal; 8 Department of Biomedicine, Faculty of Medicine of the University of Porto, Porto, Portugal

**Keywords:** follicular fluid, glycolysis, granulosa cells, metabolism, mitochondria, obesity, PCOS

## Abstract

**Background:**

Polycystic ovary syndrome (PCOS) is characterized by altered follicular development and metabolic dysfunction, frequently exacerbated by obesity. The follicular fluid (FF) microenvironment plays a critical role in supporting oocyte maturation and granulosa cell function; however, the extent to which FF from women with PCOS and obesity is associated with alterations in granulosa cell metabolism remains unclear. This study aimed to evaluate how does the FF from women with PCOS and/or obesity shapes granulosa cell glycolytic and mitochondrial activity.

**Results:**

FF from women with PCOS showed significantly increased concentrations of total testosterone and Δ4-androstenedione compared with controls, irrespective of BMI (p < 0.05 and p < 0.01, respectively). Exposure of human granulosa cell line (HGrC1) to FF from women with PCOS and obesity was associated with a marked reduction in glycolytic capacity (p < 0.05) and decreased mRNA expression of key glycolytic regulators, including GLUT1, HK2 and LDHA (p < 0.05). Mitochondrial function was also altered, as evidenced by reduced maximal respiration and mitochondrial membrane potential (p < 0.05), while reactive oxygen species levels remained unchanged. Metabolomic profiling revealed elevated glucose concentrations in FF from women with PCOS and obesity compared with normal-weight controls, consistent with potential alterations in glucose metabolism within the follicular environment.

**Conclusion:**

Granulosa cells depict metabolic dysregulation, with reduced glycolytic activity and impaired mitochondrial function, when exposed to FF from women with PCOS, which is further exacerbated in the presence of obesity. These findings from a pilot hypothesis-generating study suggest that the intrafollicular environment may be associated with granulosa cell metabolic disturbances, which warrant mechanistic studies to establish causality and elucidate the downstream consequences for follicular maturation and ovulatory function.

## 1 Introduction

Polycystic ovary syndrome (PCOS) is one of the most common endocrine disorders among women of reproductive age and the leading cause of anovulatory infertility (1–3). PCOS presents a heterogeneous spectrum of clinical and biochemical features, including hyperandrogenism, ovulatory dysfunction, and polycystic ovarian morphology (4). PCOS etiology is unknown, although it seems to derive from multifactorial complex interactions of genetic, epigenetic, developmental, and environmental factors (5–7), which shape the different PCOS phenotypes (8, 9).

Obesity and insulin resistance are also highly prevalent conditions in the general population, and when these occur alongside PCOS, reproductive and metabolic dysfunctions can both be intensified which complicates clinical management (10–12). Compelling evidence show that excessive adiposity exacerbates the vicious cycle of insulin resistance, adipose tissue dysfunction, and androgen excess in women with PCOS (10–13). Moreover, women with PCOS associated with obesity often exhibit reduced fertility and lower success rates with assisted reproductive treatments (ART) (14).

Within the follicle, the FF reflects a specialized microenvironment critical for oocyte growth and maturation (15). The FF originates from the transudation of plasma components and the secretions of granulosa and theca cells, which form the follicle wall and collectively establish a dynamic microenvironment within the follicle (15, 16). It contains a complex mixture of proteins, metabolites, lipids, hormones, and signaling molecules, reflecting both local ovarian dynamics and systemic influences (17). In women with PCOS, FF has been consistently reported to exhibit a distinct biochemical and metabolic composition compared with that of unaffected women. This altered FF profile is characterized by disruptions in lipid, amino acid, and energy metabolism, and is frequently accompanied by increased oxidative stress and a pro-inflammatory milieu (18–23). In parallel, granulosa cells (GCs) from women with PCOS also exhibited metabolic dysfunction, including impaired glycolytic and mitochondrial activity (24–26). Overall, disturbances in cellular bioenergetics are a hallmark of PCOS, contributing to follicular arrest and impaired fertility; however, it remains unclear to what extent the altered composition of FF is the cause or consequence of GCs metabolic abnormalities.

In this study, we aimed to elucidate the extent to which the FF composition from women with PCOS, with and without obesity, could contribute to GCs metabolic dysfunction. We further hypothesized that obesity acts as a metabolic modifier within the PCOS phenotype, leading to distinct and exacerbated metabolic adaptations in GCs. By assessing potential influence of FF composition on glycolytic flux and mitochondrial function, we sought to identify phenotype-specific metabolic adaptations and how obesity modulates the metabolic response of GCs within the PCOS context. This approach provides novel insights into the crosstalk between the systemic metabolic status and ovarian cellular metabolism, including how obesity may exacerbate the metabolic dysfunctions that underlie impaired follicular development and reproductive outcomes in women with PCOS.

## 2 Materials and methods

### 2.1 Study participants

A total of 24 women undergoing controlled ovarian stimulation for oocyte donation or *in vitro* fertilization (IVF) for infertility treatment were recruited to participate in this study at a Centro Materno-Infantil do Norte Albino Aroso (CMIN, Porto, Portugal) for assisted medical reproduction between January 2022 and February 2024. The diagnosis of PCOS was based on the 2003 Rotterdam diagnostic criteria, which requires the presence of at least two of the following three manifestations: (1) oligo- and/or anovulation, (2) clinical and/or biochemical evidence of hyperandrogenism, and (3) polycystic ovaries on ultrasound examination (at least 10 follicles 2–9 mm in size or volume of the ovary greater than 10 mL) (4). The control group included women who underwent ART treatments for oocyte donation or infertility treatment due to fallopian tube obstruction and/or male factors. Exclusion criteria included women with diminished ovarian reserve, endometriosis, abnormal prolactin levels, and/or thyroid dysfunction. All participants included in this study, although previously diagnosed with PCOS at referral, underwent a thorough phenotypic characterization before study enrollment. This evaluation was independently performed by two experienced physicians from the research team, specialist consultants in Reproductive Gynecology (EVF) and Endocrinology (MPM). Based on diagnosis and body mass index (BMI), participants were allocated into four different groups: normal-weight controls (n=6; BMI < 25 kg/m^2^); obesity controls (n=6; BMI ≥ 30 kg/m^2^); normal-weight PCOS (n=6; BMI < 25 kg/m^2^); obesity PCOS (n=6; BMI ≥ 30 kg/m^2^). Electronic medical records were used for data extraction, namely age, hormonal measurements, and IVF cycle characteristics. All women were recruited and enrolled in the study after providing written informed consent to participate. The study protocol was authorized by the Ethics Committee of the Institution [2020.119(097-DEFI/099-CE)]. All women underwent a Gonadotropin-Releasing Hormone (GnRH) antagonist protocol with individualized controlled ovarian stimulation (COS) based on ovarian reserve testing according to standard clinical practice. In women with PCOS, the use of a GnRH antagonist protocol combined with GnRH agonist triggering is a well-supported strategy to mitigate Ovarian Hyperstimulation Syndrome (OHSS) risk (27).

### 2.2 Peripheral blood and follicular fluid collection

Peripheral blood samples were obtained from each woman, and hormonal evaluation was performed at three different timepoints. The first assessment was conducted up to six months before the COS for IVF, within the 3^rd^ to 5^th^ day of the menstrual cycle (estradiol (E_2_), follicle-stimulating hormone (FSH), luteinizing hormone (LH), and anti-Müllerian hormone (AMH)). The second assessment was conducted after COS on the day of trigger administration for oocyte maturation (E_2_ and progesterone); and the final assessment was performed before ovarian puncture for oocyte retrieval (glucose, insulin, Δ4-androstenedione, testosterone and sex hormone-binding globulin (SHBG)). During this procedure, each follicle was individually aspirated and the FF from multiple follicles was combined into a single pool of each woman. The pooled FF was centrifuged at 20,000× *g* for 10 min to remove cells and debris. The resulting supernatant was carefully collected and aliquoted for measuring hormone levels (Δ4-androstenedione, testosterone, SHBG, E_2_ and progesterone) or stored at -80 °C until further use.

Hormone levels in plasma and FF were analyzed at the core laboratory of the public hospital where our ART center is located. Capillary blood glucose was measured using a glucometer at point of care (Freestyle Precision Neo Glucose meter, Abbott, USA). Total testosterone, Δ4-androstenedione, E_2_, progesterone, and insulin were measured using electrochemiluminescence immunoassay (ECLIA, Roche Diagnostics, Mannheim, Germany). SHBG was analyzed with a chemiluminescent immunometric assay (Immulite XPi instrument, Siemens Healthcare Diagnostics, UK). AMH levels were determined using an enzyme-linked immunosorbent assay (Beckman Coulter Access AMH, Immunotech, France).

### 2.3 Untargeted proton nuclear magnetic resonance analysis of follicular fluid metabolome

A total of 225 µL of thawed FF was mixed with cold methanol (1:2 *v/v*) for protein precipitation. After 20 min of incubation at -20 °C, samples were centrifuged at 13,000× *g* for 30 min. The supernatant was collected and dried in a vacuum concentrator 5301 (Eppendorf, Hamburg, Germany). Samples were then resuspended in 225 µL of a phosphate-buffered (40 mM, pH 6.8) sodium fumarate (2 mM) in deuterium oxide (99.9%) solution and pipetted into 3 mm NMR tubes (Norell, Landisville, NJ, USA). Spectra were acquired using NOR5X3INSB optimizer inserts (Norell, Landisville, NJ, USA) on a 600 MHz Bruker Avance III HD spectrometer equipped with a 5 mm CPP BBO 600S3 BB-H F-D-05 Z probe (Bruker Corporation, Billerica, MA, USA) at 298K. Solvent-suppressed ^1^D-^1^H-NMR spectra were acquired using a zgpr pulse sequence, with a spectral width of 12 ppm, relaxation delay of 7 s, pulse angle of 30°, acquisition time of 2.34 s and 32 scans. A line broadening of 0.2 Hz and Fourier transform were applied to the spectra. The spectra were manually phased and baseline corrected. Spectra were referenced to fumarate signal (2 mM; 6.50 ppm). Peaks were assigned through comparison with reference spectra using Chenomx (Chenomx Inc. Edmonton, Canada) and the Human Metabolome Database (HMDB) V 5.0 (28). This study is available at the NIH Common Fund’s National Metabolomics Data Repository (NMDR) website, the Metabolomics Workbench.

### 2.4 Cell culture

Human nonluteinized granulosa cell line (HGrC1), a generous gift from Dr. Ikara Iwase (Nagoya University, Japan), was used as an *in vitro* model for this study. HGrC1 retains key granulosa cell features, including expression of FSH receptor and steroidogenic enzymes, as well as responsiveness to granulosa-specific stimuli, and therefore has been widely used as an *in vitro* model of human GCs (29). HGrC1 was cultured in phenol red-free DMEM:F12 (D2906, Sigma-Aldrich, St. Louis, Missouri, USA) supplemented with 10% fetal bovine serum (FBS, Sigma Aldrich, St. Louis, Missouri, USA), 50 µg/mL gentamicin, and 1% Penicillin-Streptomycin. The culture medium was replaced every 48 h, and cells were passaged upon reaching 70–80% confluence to maintain viability and consistency.

### 2.5 Experimental groups

HGrC1 were maintained in standard culture conditions until 60–70% confluence. Ten to twelve hours before treatment, cells were serum-starved in phenol red–free DMEM:F12 medium without FBS. The FF from participants assigned to each group was mixed in equal volumes to yield a pooled FF sample representative of the respective experimental group: (1) controls with normal-weight (BMI < 25 kg/m^2^); (2) controls with obesity (BMI ≥ 30 kg/m^2^); (3) PCOS with normal-weight (BMI < 25 kg/m^2^); and (4) PCOS with obesity (BMI ≥ 30 kg/m^2^). Each pooled FF was processed as previously described (section 2.2) and filtered (0.22 µm) prior to use. For treatment, starved HGrC1 cells were incubated for 24 h in phenol red–free DMEM:F12 medium supplemented with 20% pooled FF representative of each experimental condition. After treatment, cells were used in or collected for subsequent experiments. For protocols requiring cell pellets, cells were detached from the culture flask with TrypLE Express enzyme (12604-021, Gibco, Thermo Fisher, Waltham, MA, USA) centrifuged, collected and stored at −80 °C until further use.

### 2.6 Total RNA extraction, cDNA synthesis and quantitative real-time PCR

Total RNA was extracted from HGrC1 cells using the Total RNA isolation kit (MB13402, NZYTech, Lisbon, Portugal), following the manufacturer’s instructions. RNA concentration and purity (A260/A280 and A260/A230) were determined with a NanoDrop spectrophotometer. cDNA was synthesized from 1 µg of total RNA using M-MuLV Reverse Transcriptase (MB08301, NZYTech, Lisbon, Portugal) and random hexamer primers, according to the manufacturer’s instructions. Quantitative real-time PCR (qPCR) was performed on CFX Connect™ Real-Time PCR Detection System (Bio-Rad) using the Speedy NZYtaq 2× Green Master Mix (MB22401, NZYTech, Lisbon, Portugal) and according to the manufacturer’s instructions. Briefly, 0.1 μg of cDNA of each sample was mixed with forward and reverse primers (10 μM) and Speedy NZYtaq 2× Green Master Mix. The volume was adjusted with RNase-free water. The protocol for qPCR was as follows: 2 min at 95 °C for polymerase activation following 5 s at 95 °C for DNA denaturation and 30 s at 58-62°C for annealing for a defined number of cycles. The protocol concluded with a melting-curve analysis (95 °C for 1 min, 55 °C for 30 s, and 95 °C for 30 s). All samples and a no-template control (negative control) were analyzed in duplicates, and amplification specificity was confirmed by the appearance of a single peak in the melting curve. Primer sequences, annealing temperatures, and the number of cycles required for the exponential phase of amplification in qPCR are listed in **Table 1**. All conditions were optimized for each set of primers. Relative mRNA expression levels were normalized to the housekeeping gene beta-2 microglobulin (*B2M*) and calculated using the 2^–ΔΔCt^ method (30).

**Table 1:** Primer sequences and qPCR conditions used to assess mRNA expression levels in human granulosa cell line (HGrC1).

Gene	Accession number	Sequence (5’-3’)	AT (°C)	Size (bp)	Cycles (n)
*B2M*	NM_004048.4	FWD: GGCTATCCAGCGTACTCCAA RVS: ACGGCAGGCATACTCATCTT	58 °C	247 bp	30
*IDH1*	NM_005896.4	FWD: ACGGAACCCAAAAGGTGACA RVS: GCCAACCCTTAGACAGAGCC	58 °C	138 bp	30
*CS*	NM_004077.3	FWD: TCCCACCAATCTACACCCCA RVS: CCAATACCGCTGCCTTCTCT	58 °C	210 bp	30
*COX10*	NM_001303.4	FWD: GCAAGTGTATGATTTGCCAGGA RVS: TGCAGTGGTACTTACAACCAGA	58 °C	79 bp	35
*LDHA*	NM_005566.4	FWD: ATGGCAACTCTAAAGGATCAGC RVS: CCAACCCCAACAACTGTAATCT	58 °C	86 bp	35
*CPT1A*	NM_001876.4	FWD: GCAGCGTTCTTTGTGACGTT RVS: AGGAGTGTTCAGCGTTGAGG	58 °C	184 bp	35
*GLS1*	NM_014905.5	FWD: TCTACAGGATTGCGAACGTCT RVS: CTTTGTCTAGCATGACACCATCT	58 °C	100 bp	35
*G6PD*	NM_001360016.2	FWD: CTACCGCATCGACCACTACC RVS: TGTTGTCCCGGTTCCAGATG	58 °C	101 bp	35
*HK2*	NM_000189.5	FWD: CGAGAGCATCCTCCTCAAGTG RVS: AGCCACAGGTCATCATAGTTCC	58 °C	164 bp	35
*GLUT1*	NM_006516.4	FWD: CCAGCTGCCATTGCCGTT RVS: GACGTAGGGACCACACAGTTGC	58 °C	99 bp	35

AT, Annealing temperature; *B2M*, Beta-2-Microglobulin; bp, base pairs; *CPT1A*, Carnitine Palmitoyltransferase 1A; *COX10*, Cytochrome C Oxidase Assembly Factor Heme A, Farnesyltransferase; *CS*, Citrate Synthase; *FWD*, Forward primer; *G6PD*, Glucose-6-Phosphate Dehydrogenase; *GLS1*, Glutaminase 1; *GLUT1*, Glucose Transporter Type 1; *HK2*, Hexokinase 2; *IDH1* – Isocitrate Dehydrogenase 1 (NADP+); *LDHA*, Lactate Dehydrogenase A; RVS, Reverse primer.

### 2.7 Mitochondrial membrane potential analysis

The mitochondrial membrane potential (MMP) was evaluated using the dye 5,5′,6,6′-tetrachloro-1,1′,3,3′-tetraethylbenzimidazolylcarbocyanine iodide (JC-1; T3168, Invitrogen™, Carlsbad, CA, USA). HGrC1 cells were seeded in a 96-well black culture plate with a clear bottom and treated as described above (n=6). Experimental conditions were performed in triplicate per plate. After treatment, cells were washed with PBS and incubated in FBS-free culture medium with JC-1 (1 μM) at 37 °C for 30 min in a dark environment. As an assay control, cells were incubated with 20% DMSO to induce MMP depolarization. Then, cells were washed with PBS, and fresh FBS-free culture medium was added. The fluorescence of JC-1 monomers (485/530 nm; excitation/emission) and J-aggregates (535/590 nm; excitation/emission) was evaluated using a Synergy™ H1 multi-mode microplate reader (BioTek, Winooski, VT, USA). The ratio between J-aggregates/monomers was calculated and used as an indicator for the MMP.

### 2.8 Detection of intracellular reactive oxygen species

The intracellular ROS production was estimated using the fluorescent probe CM-H_2_DCFDA (C6827, Invitrogen™, Carlsbad, CA, USA), according to the manufacturer’s instructions. HGrC1 cells were seeded in a 96-well black culture plate with a clear bottom and treated as described above (n=6). Experimental conditions were performed in triplicate per plate. Following treatment, cells were incubated with CM-H_2_DCFDA working solution (5 μM in PBS) containing 1 μL/mL of Hoechst 33342 (10 mg/mL Solution in Water, H3570, Thermo Fischer Scientific, Waltham, MA, USA) for 30 min at 37 °C. A working solution supplemented with 0.1% H_2_O_2_ was used as positive control. CM-H_2_DCFDA (495/529 nm; Ex/Em) and Hoechst 33342 (350/461 nm; Ex/Em) fluorescence was read on a Synergy™ H1 multi-mode microplate reader (BioTek, Winooski, VT, USA). Results were normalized for cell nuclei content (Hoechst 33342) and expressed in DCF Fluorescence Units.

### 2.9 Mitochondrial oxygen consumption rate and extracellular acidification rate analysis

Glycolytic and mitochondrial function in HGrC1 cells were evaluated using the Seahorse XFe24 Analyzer (Agilent Technologies, Santa Clara, CA, USA) with the Glycolytic Stress and Cell Mito Stress Test Kits, following the manufacturer’s protocols. Before each assay, HGrC1 cells were seeded in XF24 microplates at 4 × 10^4^ cells/well and cultured to reach 80% confluence. Then cells were treated under the designated experimental conditions as previously described (n = 6; each condition assayed in duplicate). Following treatment, cells were washed with PBS, and Seahorse XF assay medium (DMEM, pH 7.4) supplemented with 1 mM pyruvate and 2 mM glutamine was added to each well. For mitochondrial stress testing, the medium was also supplemented with 10 mM glucose, whereas glucose was absent in the glycolytic stress test medium to allow subsequent glucose-stimulated acidification measurements. Plates were equilibrated with assay medium for 45 min at 37 °C in a CO_2_−free incubator before measurements.

For glycolytic flux analysis, extracellular acidification rate (ECAR) was recorded under basal, glucose−starved conditions, followed by sequential injections of glucose (10 mM), oligomycin (1 µM) and 2−deoxyglucose (50 mM). Three ECAR measurements were taken at 8 min intervals between each injection over a total run time of 100–120 min. ECAR values were normalized to total protein per well using a standard BCA assay (Pierce™ BCA Protein Assay Kit, Thermo Fisher Scientific). Glycolysis, glycolytic capacity, and glycolytic reserve were calculated as the differences between key ECAR phases before and after each injection, as specified by the assay guidelines (31). Briefly, glycolysis was defined as the increase in ECAR following glucose injection, reflecting basal glycolytic activity. Glycolytic capacity was determined as the maximal ECAR reached after inhibition of mitochondrial ATP production with oligomycin, representing the cell’s maximum glycolytic flux. Glycolytic reserve was calculated as the difference between glycolytic capacity and basal glycolysis, indicating the ability of cells to upregulate glycolysis in response to energetic stress.

For mitochondrial respiration analysis, oxygen consumption rate (OCR) was measured following sequential injections of oligomycin (1.5 µM), FCCP (1 µM), and rotenone/antimycin A (0.5 µM each). As in the glycolytic assay, three OCR measurements were collected at 8 min intervals between injections over 100–120 min. From the collected data, basal respiration, proton leak, maximal respiration, spare respiratory capacity, ATP production-coupled respiration, and non-mitochondrial oxygen consumption were calculated. Basal respiration was defined as the OCR under baseline conditions, reflecting routine mitochondrial activity. ATP production - coupled respiration was calculated as the decrease in OCR following oligomycin injection and represents oxygen consumption linked to ATP synthesis. Proton leak was determined as the residual OCR after oligomycin injection, reflecting mitochondrial membrane permeability independent of ATP production. Maximal respiration was defined as the OCR achieved after FCCP injection, indicating the maximal capacity of the electron transport chain. Spare respiratory capacity was calculated as the difference between maximal and basal respiration, reflecting the ability of cells to respond to increased energetic demand. Non-mitochondrial oxygen consumption was determined as the residual OCR after inhibition of the electron transport chain and reflects oxygen consumption by non-mitochondrial processes. OCR was likewise normalized to total protein per well using a standard BCA assay (32).

### 2.10 Statistical analysis

All statistical analyses were performed using GraphPad Prism version 8.0 software (San Diego, CA, USA). Data normality was assessed using the Shapiro–Wilk test. When the data followed a normal distribution, one-way analysis of variance (ANOVA) was conducted to compare differences among groups, using the normalweight control group (BMI < 25 kg/m2) as the reference. Dunnett's post-hoc test was applied for multiple comparisons. When normality was not assumed, the Kruskal– Wallis test was used as a non-parametric alternative, followed by Dunn’s post-hoc test. For HGrC1 cell experiments, results are presented as Tukey’s box plots (median, 25th to 75th percentiles ± 1.5 IQR). For women characteristics and metabolomic profiling data, results are expressed as mean ± standard deviation (SD). A pvalue < 0.05 was considered statistically significant.

## 3 Results

### 3.1 The clinical and hormonal profile of women according to phenotype

Participants clinical data are described in **Table 2**. Age-matched women undergoing IVF were divided into 4 groups, based on BMI and PCOS diagnosis. Comparing to their normal-weight counterparts, women with obesity exhibited significantly lower levels of basal E_2_ (*p* < 0.05).

**Table 2:** Demographic characteristics and baseline hormone levels of women with polycystic ovary syndrome (PCOS) and control women, stratified by body mass index (BMI).

Participant parameters	Control BMI<25 kg/m^2^ (n=6)	Control BMI ≥30 kg/m^2^ (n=6)	PCOS BMI<25 kg/m^2^ (n=6)	PCOS BMI ≥30 kg/m^2^ (n=6)	P-value
Age (years)	35.50 ± 3.51	34.17 ± 5.27	32.17 ± 4.07	31.33 ± 3.56	NS
BMI (kg/m^2^)	21.53 ± 1.11	**33.15 ± 2.38^a^**	22.17 ± 1.90	**35.43 ± 4.94^c^**	^a^p<0.0001 ^c^p<0.0001
Baseline hormone levels					
E_2_ (pg/mL)	65.84 ± 19.51	**35.86 ± 17.78^a^**	48.33 ± 20.78	**31.90 ± 15.62^c^**	^a^p<0.05 ^c^p<0.05
LH (mIU/mL)	5.52 ± 1.32	5.02 ± 2.48	**19.53 ± 6.48^b^**	8.93 ± 3.67	^b^p<0.0001
FSH (mIU/mL)	8.18 ± 2.81	5.80 ± 2.25	7.78 ± 2.00	5.65 ± 1.46	NS
LH: FSH ratio	0.72 ± 0.20	0.82 ± 0.32	**2.65 ± 1.24^b^**	1.60 ± 0.63	^b^p<0.001
Glucose (mg/dL) *	93.00 ± 3.39	93.60 ± 11.41	88.20 ± 10.28	89.20 ± 12.60	NS
Insulin (μIU/mL) *	7.86 ± 2.11	13.71 ± 7.07	4.90 ± 2.00	15.06 ± 6.32	NS
HOMA-IR	1.81 ± 0.54	3.28 ± 1.85	1.04 ± 0.47	3.24 ± 1.26	NS
AMH (pmol/L)	13.37 ± 3.79	22.95 ± 6.42	**79.72 ± 67.26^b^**	52.63 ± 29.71	^b^p<0.05

Values are presented as mean ± standard deviation. A *p*-value < 0.05 was considered statistically significant. AMH, anti-Müllerian hormone; BMI, body mass index; E_2_, β2-estradiol; FSH, follicle-stimulating hormone; HOMA-IR, Homeostatic Model Assessment for Insulin Resistance; IU, international units; LH, luteinizing hormone; NS, no significance; PCOS, polycystic ovary syndrome; a denotes a statistically significant difference (*p* < 0.05) between control BMI<25 kg/m^2^ compared with control BMI ≥30 kg/m^2^; b denotes a statistically significant difference (*p* < 0.05) between control BMI<25 kg/m^2^ compared with PCOS BMI<25 kg/m^2^; c denotes a statistically significant difference (*p* < 0.05) between control BMI<25 kg/m^2^ compared with PCOS BMI ≥30 kg/m^2^. The values in bold indicate statistically significant results.

Additionally, normal-weight women with PCOS presented significantly higher levels of LH (*p* < 0.0001) as well as a higher LH: FSH ratio (*p* < 0.001) as compared to normal-weight controls. In contrast, although women with PCOS and obesity also exhibited numerically higher LH concentrations and LH:FSH ratios, these differences did not reach statistical significance compared to normal-weight controls. A similar pattern was observed for AMH levels, as normal-weight women with PCOS demonstrated significantly higher AMH levels as compared to normal-weight controls (*p* < 0.05), but AMH levels of women with PCOS and obesity did not reach statistical difference (*p* = 0.23) (**Table 2**).

Metabolic parameters, including fasting glucose and insulin levels, did not present statistically significant differences across the study groups. However, women with obesity, regardless of PCOS status, exhibited clinically relevant higher HOMA−IR indices (>3), which is within the insulin resistance range.

On the day of oocyte retrieval, women with PCOS and obesity exhibited significantly higher plasma levels of Δ4-androstenedione (*p* < 0.05) and lower SHBG (*p* < 0.05) compared to the normal-weight control group. Additionally, this group showed a significantly increased free androgen index (FAI) (*p* < 0.01), further indicating a state of androgen excess relative to normal-weight controls (**Table 3**).

**Table 3:** Biochemical characteristics of plasma and Follicular Fluid (FF) on the day of oocyte retrieval in women with polycystic ovary syndrome (PCOS) andÜontrol women, stratified by body mass index (BMI).

Participant parameters	Control BMI<25 kg/m^2^ (n=6)	Control BMI ≥30 kg/m^2^ (n=6)	PCOS BMI<25 kg/m^2^ (n=6)	PCOS BMI ≥30 kg/m^2^ (n=6)	P-value
Oocyte retrieval day- plasma levels				
Δ4-androstenedione (ng/mL)	2.85 ± 0.43	2.88 ± 0.80	4.37 ± 1.97	**7.13 ± 5.50^c^**	^c^p<0.05
Testosterone (ng/mL)	1.01 ± 0.23	1.33 ± 0.63	1.45 ± 0.65	2.61 ± 2.10	NS
SHBG (nmol/L)	203.00 ± 38.91	131.80 ± 32.03	146.50 ± 72.17	**116.30 ± 54.48^c^**	^c^p<0.05
FAI (%)	1.83 ± 0.64	3.93 ± 2.42	4.11 ± 2.39	**7.54 ± 2.95^c^**	^c^p<0.01
Oocyte retrieval day- follicular fluid levels				
Δ4-androstenedione (ng/mL)	5.53 ± 3.45	7.48 ± 2.95	**77.87 ± 53.46^b^**	**119.9 ± 59.42^c^**	^b^p<0.05 ^c^p<0.001
Testosterone (ng/mL)	5.77 ± 1.83	6.99 ± 2.51	**25.23 ± 13.52^b^**	**30.18 ± 19.77^c^**	^b^p<0.05 ^c^p<0.01
SHBG (nmol/L)	213.70 ± 85.92	77.08 ± 25.73	220.20 ± 158.80	95.03 ± 42.90	NS
Progesterone (ng/mL)	14481 ± 4040	14691 ± 6708	9998 ± 2514	8522 ± 5256	NS
Estradiol (pg/mL)	395333 ± 111826	351467 ± 142448	712533 ± 417410	819133 ± 512039	NS

Values are presented as mean ± standard deviation. A p-value < 0.05 was considered statistically significant. FAI, free androgen index; NS, no significance; PCOS, polycystic ovary syndrome; SHBG, sex hormone binding globulin. a denotes a statistically significant difference (*p* < 0.05) between control BMI<25 kg/m^2^ compared with control BMI ≥30 kg/m^2^; b denotes a statistically significant difference (*p* < 0.05) between control BMI<25 kg/m^2^ compared with PCOS BMI<25 kg/m^2^; c denotes a statistically significant difference (*p* < 0.05) between control BMI<25 kg/m^2^ compared with PCOS BMI ≥30 kg/m^2^. The values in bold indicate statistically significant results.

The Δ4-androstenedione levels in the FF were also significantly higher in both PCOS groups, normal-weight (*p* < 0.05) and obesity (*p* < 0.001), compared to normal-weight controls. Similarly, total testosterone levels in the FF were significantly higher in the PCOS groups, regardless of BMI: in normal-weight women (*p* < 0.05) and in those with obesity (*p* < 0.01), compared to normal-weight controls (**Table 3**).

### 3.2 Follicular fluid of women with PCOS and obesity contains higher levels of glucose

To explore the metabolic composition of the follicular microenvironment, the FF metabolome was analyzed using ^1^H-NMR spectroscopy, allowing the identification and quantification of 12 metabolites (**Table 4**). Among these, glucose and threonine concentrations differed significantly between subgroups. Specifically, FF glucose levels were higher in women with PCOS and obesity relative to normal−weight controls (*p* < 0.05). Threonine concentrations were higher in both obesity groups: controls with obesity (*p* < 0.05) and PCOS with obesity (*p* < 0.05), as compared to normal−weight.

**Table 4:** Metabolite concentration (mM) in Follicular Fluid (FF) of women with polycystic ovary syndrome (PCOS) and normoovulatory control women, stratified by body mass index (BMI).

Metabolite (multiplicity, chemical shift)	Concentration (mM)				*P*-value
	Control BMI<25 kg/m^2^(n=6)	Control BMI ≥30 kg/m^2^(n=6)	PCOS BMI<25 kg/m^2^(n=6)	PCOS BMI ≥30 kg/m^2^(n=6)	
Acetate (s, 1.91 ppm)	0.16 ± 0.02	0.15 ± 0.02	0.16 ± 0.02	0.16 ± 0.02	NS
Choline (s, 3.19 ppm)	0.05 ± 0.01	0.08 ± 0.04	0.05 ± 0.01	0.07 ± 0.02	NS
D-Glucose* (d, 5.21 ppm)	2.11 ± 0.43	1.95 ± 0.65	2.28 ± 0.71	**2.94 ± 0.42^c^**	^c^p<0.05
Formate (s, 8.44 ppm)	0.04 ± 0.01	0.04 ± 0.01	0.05 ± 0.01	0.05 ± 0.02	NS
Glycine (s, 3.54 ppm)	0.34 ± 0.07	0.34 ± 0.04	0.32 ± 0.08	0.30 ± 0.08	NS
Lactate (d, 1.32 ppm)	3.16 ± 0.59	3.97 ± 1.34	2.93 ± 1.37	2.82 ± 0.60	NS
L-Alanine (d, 1.47 ppm)	0.36 ± 0.06	0.39 ± 0.07	0.38 ± 0.09	0.38 ± 0.10	NS
L-Glutamine (m, 2.45 ppm)	0.33 ± 0.05	0.31 ± 0.09	0.35 ± 0.11	0.30 ± 0.07	NS
L-Isoleucine (d, 1.00 ppm)	0.10 ± 0.02	0.10 ± 0.03	0.10 ± 0.03	0.10 ± 0.02	NS
L-Leucine (t, 0.95 ppm)	0.10 ± 0.02	0.11 ± 0.02	0.11 ± 0.01	0.11 ± 0.02	NS
L-Threonine (m, 4.24 ppm)	0.23 ± 0.03	**0.28 ± 0.05^a^**	0.26 ± 0.02	**0.29 ± 0.04^c^**	^a^p<0.05 ^c^p<0.05
L-Valine (d, 0.98 ppm)	0.20 ± 0.04	0.22 ± 0.05	0.22 ± 0.02	0.22 ± 0.05	NS

Values are presented as mean ± standard deviation (SD). A *p*-value < 0.05 was considered statistically significant. D-glucose* concentration was calculated based on α-D-glucose quantification assuming a 40% α-anomer fraction of total glucose. s, singlet; d, doublet; t, triplet; m, multiplet; NS, no significance; PCOS, polycystic ovary syndrome. a denotes a statistically significant difference (*p* < 0.05) between control BMI<25 kg/m^2^ compared with control BMI ≥30 kg/m^2^; b denotes a statistically significant difference (*p* < 0.05) between control BMI<25 kg/m^2^ compared with PCOS BMI<25 kg/m^2^; c denotes a statistically significant difference (*p* < 0.05) between control BMI<25 kg/m^2^ compared with PCOS BMI ≥30 kg/m^2^. The values in bold indicate statistically significant results.

### 3.3 Follicular fluid from women with PCOS and obesity modulates the expression of enzymes involved in glucose uptake, glycolysis and fatty acid β-oxidation in granulosa cells

**Figure 1:**
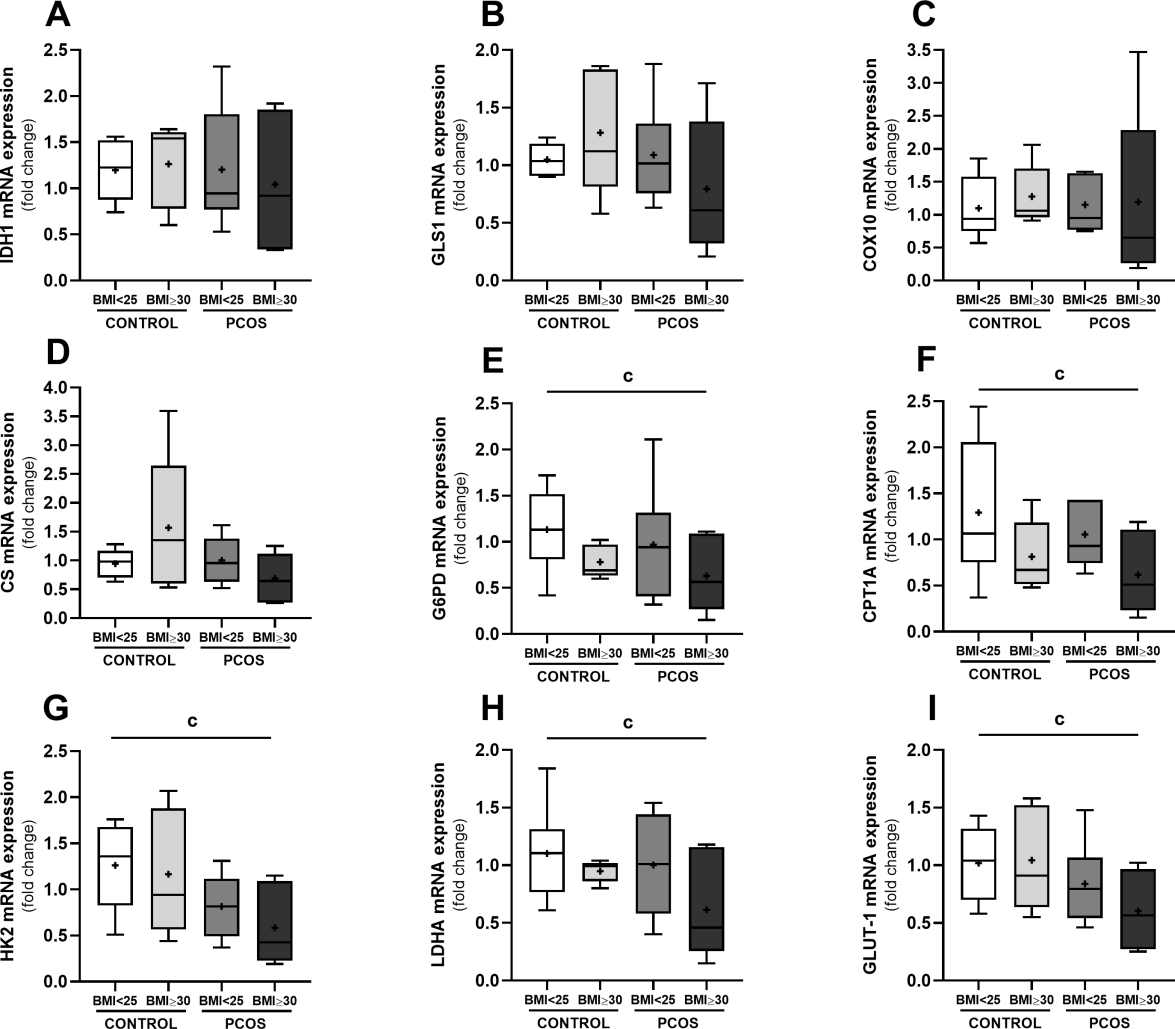
Relative mRNA expression of key enzymes involved in cellular energy metabolism in HGrC1 cells after follicular fluid supplementation (20%) for 24h (n=6, duplicates for each condition). *IDH1* [Panel **(A)**], *GLS1* [Panel **(B)**], *COX10* [Panel **(C)**], *CS* [Panel **(D)**], *G6PD* [Panel **(E)**], *CPT1A* [Panel **(F)**], *HK2* [Panel **(G)**], *LDHA* [Panel **(H)**], *GLUT1* [Panel **(I)**]. Results are expressed as Tukey’s boxplot (median, 25th to 75th percentiles ± 1.5 IQR). + represents group average; c denotes a statistically significant difference (*p* < 0.05) between control BMI<25 kg/m^2^ compared with PCOS BMI ≥30 kg/m^2^. *COX10*, cytochrome c oxidase subunit 10; *CPT1A*, carnitine palmitoyltransferase 1A; *CS*, citrate synthase; *G6PD*, glucose-6-phosphate dehydrogenase; *GLS1*, glutaminase 1; *GLUT1*, glucose transporter 1; *HK2*, hexokinase 2; *IDH1*, isocitrate dehydrogenase 1; *LDHA*, lactate dehydrogenase.

To identify putative metabolic pathways that are influenced by the molecular profile of the FF, we conducted a targeted analysis of key enzymes involved in cellular energy metabolism in GCs. Quantitative real-time PCR was used to assess the transcript levels of enzymes related to glucose uptake, glycolysis, the pentose phosphate pathway, fatty acid β-oxidation, and the tricarboxylic acid (TCA) cycle (**Figure 1**). The analyzed enzymes included glucose transporter 1 (GLUT1); hexokinase 2 (HK2) and lactate dehydrogenase A (LDHA); glucose-6-phosphate dehydrogenase (G6PD); carnitine palmitoyltransferase 1A (CPT1A); citrate synthase (CS) and isocitrate dehydrogenase 1 (IDH1); glutaminase 1 (GLS1); and cytochrome c oxidase subunit 10 (COX10).

GCs exposed to FF from women with PCOS and obesity exhibited a marked downregulation of key genes in glucose uptake and glycolysis. Specifically, *GLUT-1* (0.61 ± 0.35), *HK2* (0.58 ± 0.43), and *LDHA* (0.62 ± 0.45) mRNA levels were reduced compared to cells treated with FF from normal−weight controls (*GLUT1*: 1.02 ± 0.32; *HK2*: 1.26 ± 0.47; *LDHA*: 1.10 ± 0.42; *p* < 0.05). Similarly, expression of *G6PD*, which supplies cytosolic NADPH for reductive biosynthesis and antioxidant defenses, was significantly reduced in cells treated with FF from women with PCOS and obesity (0.63 ± 0.42) compared with normal-weight controls (1.13 ± 0.45; *p* < 0.05). In parallel, *CPT1A*, which facilitates mitochondrial import of long-chain fatty acids for β-oxidation, was also downregulated in cells exposed to FF from women with PCOS and obesity to 0.62 ± 0.45 compared to those treated with FF from normal-weight controls (1.29 ± 0.76, *p* < 0.05), suggesting a diminished capacity for fatty-acid oxidation. On the other hand, transcript levels of *IDH1, COX10, CS*, and *GLS1* did not present significant differences among groups.

These findings demonstrate that FF from women with PCOS and obesity exerts a suppressive effect on key metabolic pathways in GCs, particularly those related to glycolysis, NADPH generation, and fatty acid β-oxidation. This metabolic reprogramming may contribute to the bioenergetic insufficiency and impaired follicular function commonly observed in women with PCOS.

### 3.4 Follicular fluid from women with PCOS and obesity impairs granulosa cell mitochondrial function

**Figure 2:**
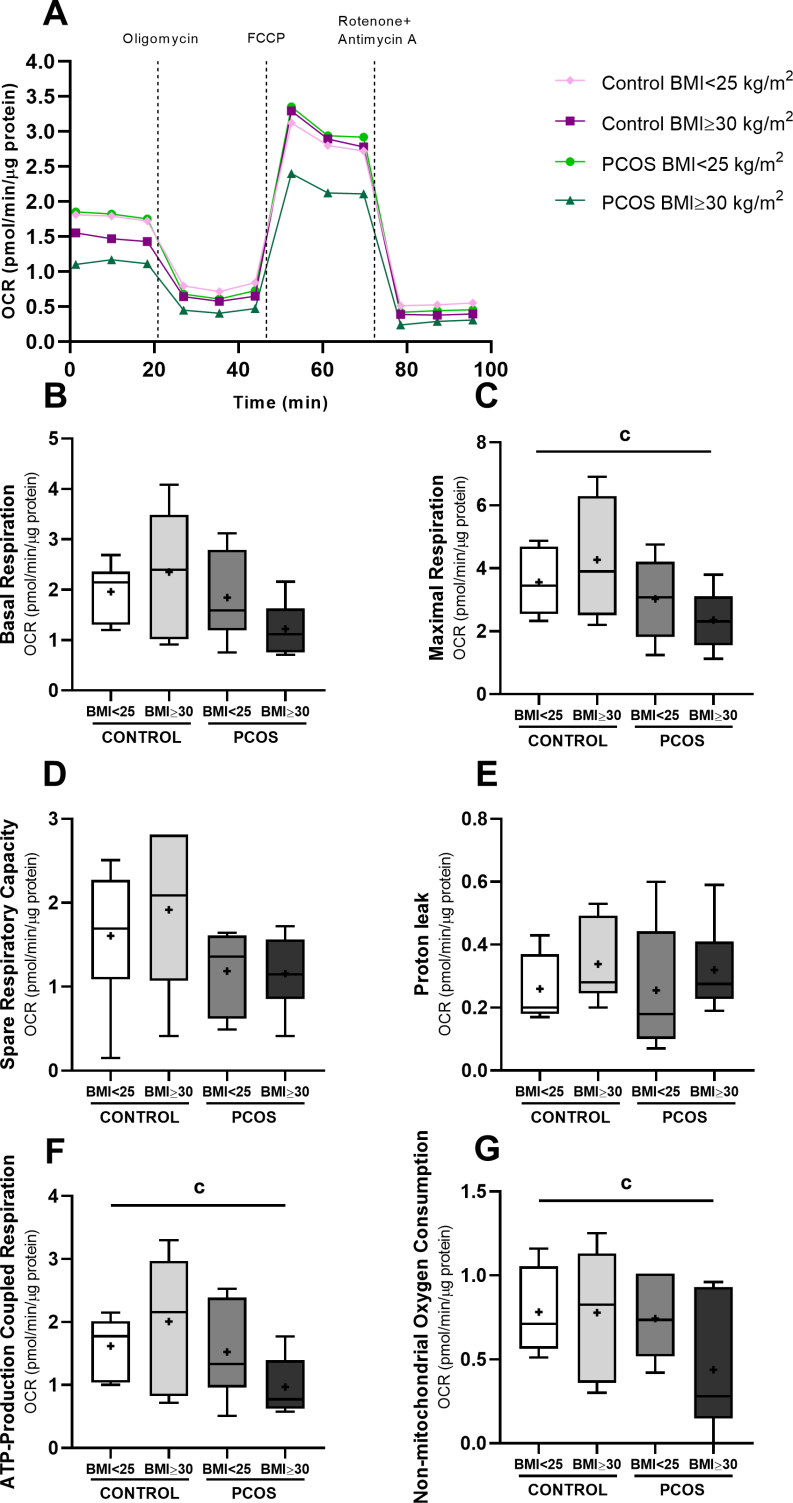
Mitochondrial respiratory function of HGrC1 cells after follicular fluid supplementation (20%) for 24h (n=6, duplicates for each condition). Representative image of oxygen consumption rate (OCR) [PANEL **(A)**], Basal respiration [Panel **(B)**], maximal respiration [Panel **(C)**], spare respiration capacity [Panel **(D)**], proton leak [Panel **(E)**], ATP-production coupled respiration [Panel **(F)**], and non-mitochondrial oxygen consumption [Panel **(G)**]. Results are expressed as Tukey’s boxplot (median, 25th to 75th percentiles ± 1.5 IQR). + represents group average; c denotes a statistically significant difference (*p* < 0.05) between control BMI<25 kg/m^2^ compared with PCOS BMI ≥30 kg/m^2^.

To investigate how FF from normoovulatory and PCOS women, with and without obesity, affects granulosa cell mitochondrial function, we performed real−time measurements of the OCR using the Seahorse XFe24 Analyzer (**Figure 2**).

Mitochondrial function was evaluated by measuring the OCR under basal conditions and following sequential injections of: (i) oligomycin (ATP synthase inhibitor) to quantify ATP-linked respiration and proton leak; (ii) FCCP (a mitochondrial uncoupler) to assess maximal respiration; and (iii) a combination of rotenone and antimycin A (Complex I and III inhibitors) to determine the non-mitochondrial respiration.

GCs treated with FF from women with PCOS and obesity exhibited a trend toward lower basal respiration (1.22 ± 0.56 pmol/min/µg protein) as compared to those treated with FF from normal-weight controls (1.96 ± 0.57 pmol/min/µg protein, *p* = 0.08). A significantly lower ATP-linked respiration was observed in cells treated with FF from women with PCOS and obesity (0.97 ± 0.46 pmol/min/µg protein) as compared to those treated with FF from normal-weight controls (1.62 ± 0.49 pmol/min/µg protein, *p* < 0.05), whereas proton leak was not significantly different among all groups. Maximal respiration was also significantly lower in the group treated with FF from women with PCOS and obesity (2.35 ± 0.93 pmol/min/µg protein) as compared to the group treated with FF from normal-weight controls (3.56 ± 1.07 pmol/min/µg protein, *p* < 0.05). Maximal respiration, defined as the highest OCR achieved following FCCP stimulation, reflects the maximum mitochondrial respiratory capacity under conditions of increased energetic demand. Therefore, the observed reduction suggests that mitochondria have a limited capacity to upregulate oxidative metabolism in response to increased energy demands. Similarly, non-mitochondrial respiration, was also significantly lower in the same FF-treated group (PCOS with obesity: 0.44 ± 0.40 pmol/min/µg protein) when compared to the group treated with FF from normal-weight control (0.78 ± 0.26 pmol/min/µg protein, p < 0.05), suggesting a global suppression of cellular oxygen‐consuming processes beyond mitochondria.

**Figure 3:**
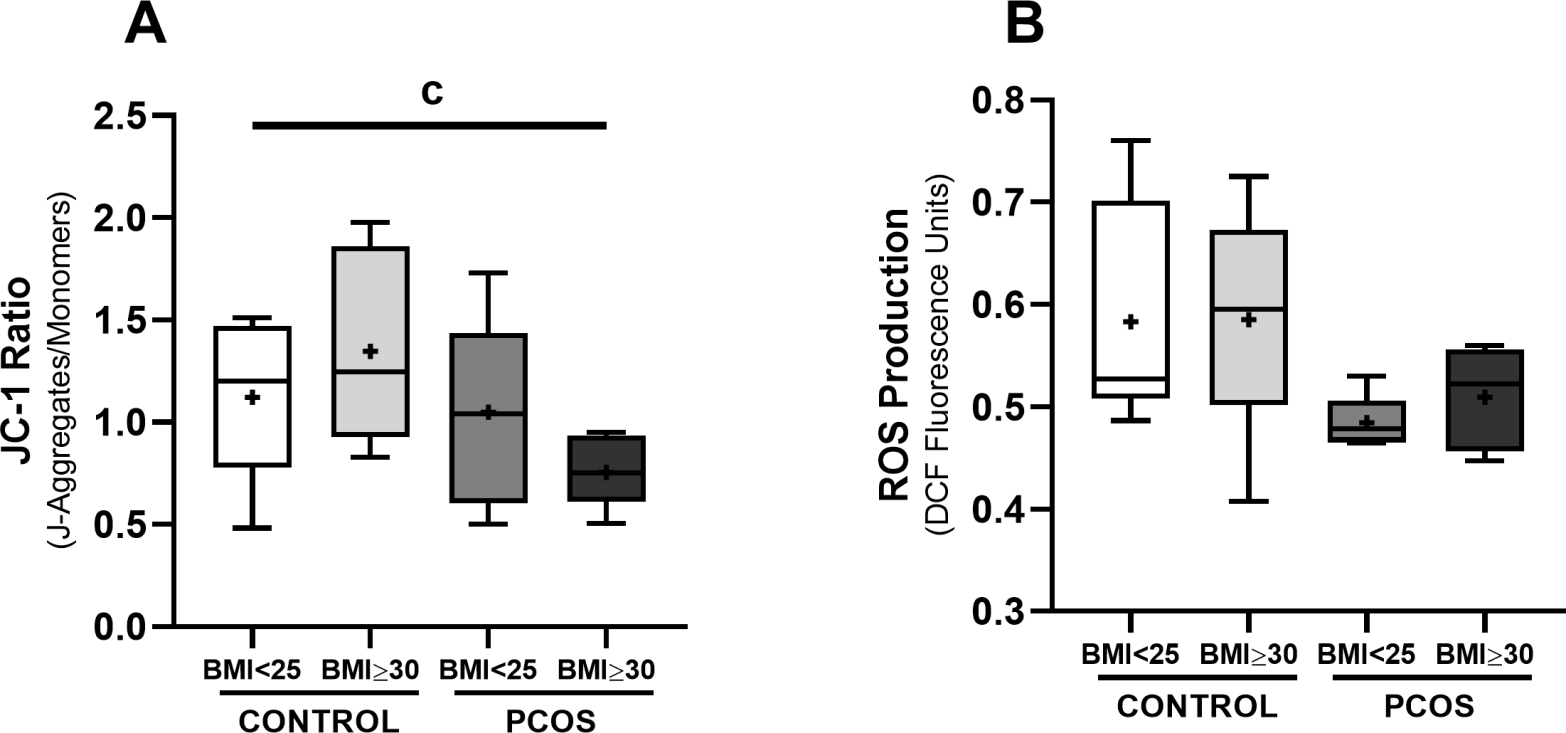
Mitochondrial membrane polarization and endogenous ROS production in HGrC1 cells after follicular fluid supplementation (20%) for 24h (n=6, duplicates for each condition). Evaluation of mitochondrial membrane potential using the JC-1 dye [Panel **(A)**], Evaluation of endogenous ROS production using the CM-H_2_DCFDA dye [Panel **(B)**]. Results are expressed as Tukey’s boxplot (median, 25th to 75th percentiles ± 1.5 IQR). + represents group average; c denotes a statistically significant difference (*p* < 0.05) between control BMI<25 kg/m^2^ compared with PCOS BMI ≥30 kg/m^2^.

Mitochondrial membrane potential was further assessed using the JC-1 fluorescent dye (**Figure 3A**). GCs treated with FF from women with PCOS and obesity showed a significantly lower JC-1 ratio (0.76 ± 0.17 J-aggregates/monomers) compared with the cells treated with FF from normal-weight controls (1.12 ± 0.39 J-aggregates/monomers, *p* < 0.05), suggesting mitochondrial depolarization and compromised mitochondrial activity. Since mitochondria are a primary source of intracellular ROS, we also evaluated the total intracellular ROS production (**Figure 3B**). No significant differences were observed in intracellular ROS among the FF-treated groups, suggesting that the observed mitochondrial dysfunction may occur independently of the oxidative stress.

Collectively, these data demonstrate that FF from women with PCOS and obesity is associated with disrupted mitochondrial bioenergetics in GCs. The observed impairments in maximal respiration, ATP-linked respiration, non-mitochondrial oxygen‐consuming pathways and mitochondrial membrane potential may contribute to reduced GCs function and compromised follicular microenvironment in women with PCOS and obesity.

### 3.5 Follicular fluid from women with PCOS and obesity impairs granulosa cell glycolytic function

**Figure 4:**
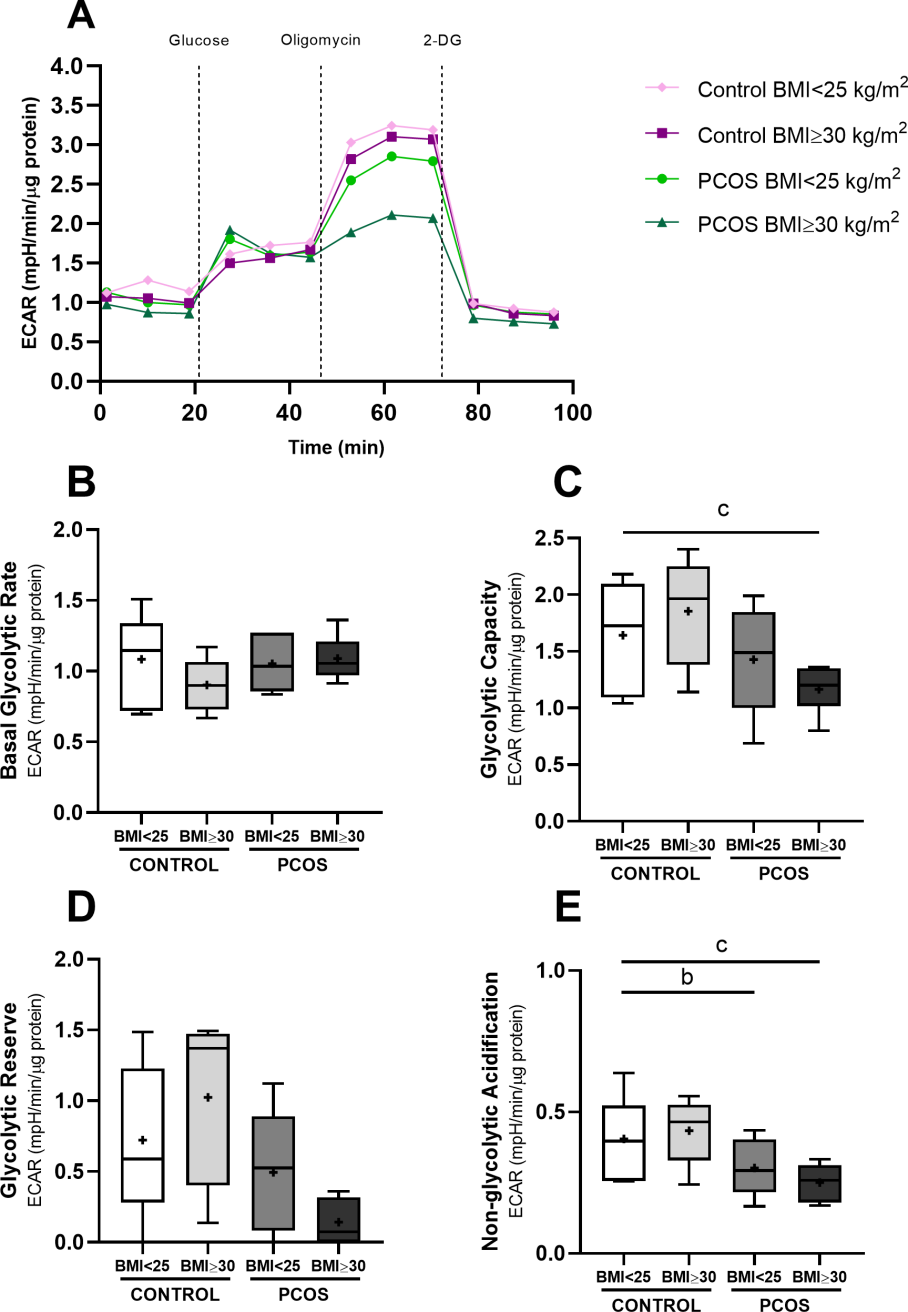
Glycolytic function of HGrC1 cells after follicular fluid supplementation (20%) for 24h (n=6, duplicates for each condition). Representative image of extracellular acidification rate (ECAR) [Panel **(A)**], basal glycolytic rate [Panel **(B)**], glycolytic capacity [Panel **(C)**], glycolytic reserve [Panel **(D)**], non-glycolytic acidification [Panel **(E)**]. Results are expressed as Tukey’s boxplot (median, 25th to 75th percentiles ± 1.5 IQR). + represents group average; b denotes a statistically significant difference (*p* < 0.05) between control BMI < 25 kg/m^2^ compared with PCOS BMI<25 kg/m^2^; c denotes a statistically significant difference (*p* < 0.05) between control BMI < 25 kg/m^2^ compared with PCOS BMI ≥30 kg/m^2^.

Human GCs rely predominantly on glycolysis, but we found that key glycolytic genes expression is lower in women with PCOS and obesity. To investigate the impact of FF from women with PCOS, with and without obesity, on GCs glycolytic function, we performed real-time extracellular acidification rate (ECAR) measurements using the Seahorse XFe24 Analyzer, which provides a dynamic assessment of glycolytic activity (**Figure 4**). Glycolytic function was evaluated by measuring ECAR in response to sequential injections of: (i) glucose to initiate glycolysis and record basal glycolytic rate; (ii) oligomycin to inhibit mitochondrial ATP synthesis and reveal maximal glycolytic capacity; and (iii) 2−DG, a competitive glucose analogue, to block glycolysis and quantify non−glycolytic acidification.

Basal glycolytic rate did not significantly differ among the FF-treated groups (**Figure 4A**). However, GCs exposed to FF from women with PCOS and obesity exhibited a significant reduction in glycolytic capacity (1.64 ± 0.48 mpH/min/μg protein) as compared to normal-weight controls (1.17 ± 0.22 mpH/min/μg protein, *p* < 0.05) (**Figure 4B**), suggesting an impaired ability to enhance glycolysis under conditions of mitochondrial stress. Glycolytic capacity corresponds to the maximal ECAR measured following inhibition of mitochondrial ATP synthesis with oligomycin and reflects the highest rate at which cells can generate ATP through glycolysis when oxidative phosphorylation is limited. Additionally, non-glycolytic acidification, defined as the residual proton production remaining after inhibition of glycolysis with 2-DG injection, was also significantly lower in GCs treated with FF from both groups of women with PCOS, normal-weight (0.30 ± 0.10 mpH/min/μg protein) and with obesity (0.25 ± 0.07 mpH/min/μg protein), as compared to the cells treated with FF from normal-weight controls (0.40 ± 0.15 mpH/min/μg protein, p<0.05)(**Figure 4D**). As non-glycolytic acidification reflects proton generation from sources other than glycolysis, such as CO_2_ hydration and amino acid catabolism, its reduction suggests decreased activity of these alternative metabolic pathways, consistent with an overall attenuation of cellular metabolic activity in GCs exposed to FF from women with PCOS. Together, these findings reveal that FF from women with PCOS, particularly in the context of obesity, compromises key aspects of GCs glycolytic function, including their capacity to meet energy demands when mitochondrial respiration is impaired. This glycolytic dysfunction may contribute to altered follicular development in PCOS.

## 4 Discussion

In this study, we investigated whether the metabolic function of GCs could be influenced by the composition of the FF from women with PCOS and obesity. Using an immortalized human granulosa cell model, we showed that exposure to FF from women with PCOS, particularly in the context of obesity, was associated with reduced glycolytic capacity and impaired mitochondrial respiration.

The mechanisms underlying follicular dysfunction in PCOS remain a major challenge to be unveiled, particularly given the heterogeneity of its clinical phenotypes. Obesity is among the most frequent comorbid conditions that affects women with PCOS, and it is known to aggravate both reproductive and metabolic manifestations. Yet, the pathways underlying ovarian dysfunction remain poorly understood. The analysis of the FF has emerged as a powerful tool to elucidate the molecular pathways contributing to follicular impairment in PCOS and to discern how distinct phenotypes, such as obesity-associated PCOS, may differentially impact granulosa cell metabolism and follicular competence. In this study, we investigated how the FF from women with PCOS, with and without obesity, modulates GC energy metabolism to uncover which interactions are PCOS-specific or obesity-related.

In our cohort, the clinical and endocrine profiles of women differed across groups according to both PCOS status and BMI. Baseline measurements showed lower circulating E_2_ in women with obesity, consistent with reports that higher adiposity is associated with lower E_2_ levels in premenopausal women (33, 34). Women with PCOS exhibited the characteristic elevation in LH and an increased LH:FSH ratio, although these alterations were attenuated in participants with concomitant obesity. This observation aligns with previous reports suggesting that obesity attenuates LH hypersecretion in PCOS (13, 35). Such modulation of hypothalamic–pituitary–ovarian (HPO) axis activity by obesity may occur through mechanisms involving insulin resistance, leptin and adipokine signaling, and altered hypothalamic–pituitary feedback (36). Furthermore, AMH concentrations were higher in women with PCOS, but this was less pronounced among women with obesity. Insulin concentrations and HOMA-IR tended to be higher in women with obesity, and although these differences did not reach statistical significance, the mean values were above 3.0, consistent with clinically relevant insulin resistance (37–40). On the oocyte retrieval day, women with PCOS, particularly those with obesity, demonstrated higher circulating Δ4-androstenedione and lower SHBG concentrations, resulting in higher FAI, which is consistent with exacerbated functional hyperandrogenism.

In the present study, HGrC1 cells exposed to FF from women with PCOS and obesity exhibited a marked suppression of glycolytic activity, as evidenced by the lower glycolytic capacity measured by ECAR and lower expression of key glycolytic genes, including *GLUT1, HK2*, and *LDHA*. These alterations suggest a reduced capacity to uptake and/or metabolize glucose efficiently, potentially reflecting changes in cellular energy metabolism. GCs are metabolically active cells that sustain oocyte growth and maturation through tight bidirectional communication (41, 42). Because oocytes have limited glycolytic capacity, they rely on GCs to metabolize glucose and provide energy substrates such as pyruvate and lactate (43, 44). Moreover, glycolytic activity in GCs has been implicated in primordial follicle recruitment and follicle growth via mTOR signaling, emphasizing its role in folliculogenesis (45). Our findings align with previous reports describing impaired glycolytic activity in GCs, though most prior studies used androgen-treated models to mimic PCOS (46–48). In our study, although both PCOS groups exhibited elevated FF testosterone and androstenedione levels compared with normal-weight controls, only HGrC1 cells treated with FF from women with PCOS and obesity depicted a significant reduction in glycolytic capacity. These observations suggest that hyperandrogenism alone may not be sufficient to influence glycolysis in GCs and that obesity-related and PCOS-specific factors could be associated with synergistic effects on glycolytic function within the follicular environment, which warrants further mechanistic investigation.

Furthermore, through ^1^H-NMR analysis, we found higher glucose concentrations in the FF of women with PCOS and obesity. This observation may be associated with alterations in glucose metabolism, which could potentially arise from reduced glucose uptake/consumption by follicular cells or from increased glucose influx from the circulation. However, because we did not observe differences in systemic glucose levels between our groups, we speculate that elevated follicular glucose concentrations may be related to altered glucose uptake or glucose metabolism; however this remains hypothetic since direct mechanistic evidence supporting this interpretation was not retrieved at the present study and warrants further investigation. This observation is consistent with previous multi-omics studies reporting that the FF of women with PCOS displays widespread alterations in metabolites related to glucose metabolism, amino acid turnover, and lipid metabolism, indicating profound dysregulation of energy pathways within the follicular microenvironment. Several of these studies have linked such metabolic disturbances to disrupted energy metabolism in GCs. Elevated branched-chain amino acid (BCAA) concentrations have been observed in FF of women with PCOS, and Mendelian randomization analyses identified PPM1K as a key regulator linking altered BCAA metabolism to PCOS traits. Consistently, PPM1K knockdown in human GCs disrupted glucose metabolism and mitochondrial function (49). Moreover, dysregulated FF–derived microRNAs have been implicated in the suppression of glycolytic enzymes such as HK2, PKM2, and LDHA, thereby reducing glycolytic flux and energy production in GCs (50, 51). In our study, we also observed higher threonine concentrations in FF from women with obesity, irrespective of PCOS status. Threonine primarily functions as a substrate for protein synthesis, and can also enter catabolic pathways, producing metabolites such as glycine, acetyl-CoA, and pyruvate, which are important for cellular and systemic metabolism (52). This observation contrasts with previous reports of lower threonine levels in the FF of women with PCOS (53, 54). To date, no mechanistic explanations have been proposed for these differences in threonine levels in the FF of women with PCOS. These inconsistencies underscore the limited understanding of threonine dynamics within the ovarian environment and highlight the need for further studies to elucidate its potential role in follicular cells’ metabolism and ovarian physiology. Despite these insights, few studies have stratified women with PCOS according to BMI, limiting the ability to distinguish metabolic disturbances intrinsic to PCOS from those arising from obesity. Our results extend current knowledge by showing that alterations in GC metabolic parameters were more pronounced in the context of coexisting PCOS and obesity, whereas FF from normal-weight women with PCOS or from control women with obesity was not associated with comparable metabolic changes.

Alongside the suppression of glycolysis, HGrC1 cells exposed to FF from women with PCOS and obesity also exhibited impaired mitochondrial function. These cells displayed significantly reduced maximal respiration and ATP-linked respiration, consistent with alterations in mitochondrial oxidative capacity. Consistently, MMP was markedly decreased, further supporting the presence of mitochondrial dysfunction in this FF-treated group. Interestingly, ROS levels did not differ among groups, suggesting that mitochondrial impairment may primarily reflect reduced bioenergetic efficiency rather than overt oxidative stress. These findings are consistent with an interdependence between glycolysis and mitochondrial activity in GCs metabolism. Mitochondria in GCs are central not only for ATP generation but also for steroid hormone biosynthesis and redox regulation. The intimate coupling between glycolysis, the tricarboxylic acid (TCA) cycle, and oxidative phosphorylation (OXPHOS) ensures that GCs meet their own energy demands while sustaining oocyte maturation. Accumulating evidence indicates that mitochondrial dysfunction is a hallmark of GCs in PCOS, encompassing abnormalities in oxidative stress balance, mitochondrial biogenesis and dynamics, and mitochondrial DNA integrity (24, 55–57). Particularly reduced MMP, lower ATP synthesis, and impaired OXPHOS have been consistently reported in GCs from women with PCOS (25, 58). Several studies have implicated reduced sirtuin activity as a key contributor to mitochondrial impairment in GCs from women with PCOS (59). Particularly SIRT3, a mitochondrial NAD^+^-dependent deacetylase, was found significantly reduced in GCs from women with PCOS, and its knockdown in KGN cells recapitulates the mitochondrial and metabolic defects observed in PCOS, including decreased ATP production, increased mitochondrial ROS, and impaired insulin (25). Additionally, SIRT3 overexpression has been shown to restore mitochondrial function and reduce apoptosis in androgen-treated GCs through activation of the FOXO1/PGC-1α signaling pathway, whereas inhibition of PGC-1α abolishes these protective effects (60). Our results reinforce the hypothesis that bioactive components within the FF may contribute to GCs’ metabolic disruption. Moreover, our data point to a synergistic interaction between PCOS-related and obesity-related factors, which together could be associated with mitochondrial dysfunction, via mechanisms that are still poorly characterized in PCOS.

We also observed a concomitant downregulation of *CPT1A*, a rate-limiting enzyme that mediates long-chain fatty acid transport into mitochondria for β-oxidation. This finding points to a further reduction in mitochondrial energy production through lipid oxidation. While systemic lipid metabolism abnormalities are documented in PCOS, particularly among women with obesity, the metabolic landscape within the ovary remains much less explored (61). Few studies have profiled lipid alterations in the FF of women with PCOS, revealing significant disturbances in lipid species composition and signaling molecules (62–64). Functionally, elevated levels of oleic acid have been shown to upregulate IL-6 and IL-8 via ERK1/2 activation in KGN cells, linking altered lipid profiles to local inflammatory signaling (65), whereas elevated levels of arachidonic acid can impair mitochondrial activity and secretory function while promoting apoptosis in GCs (66). Taken together, our results extend previous evidence by suggesting that the combination of PCOS-related and obesity-related lipotoxicity may jointly disrupt the balance between glycolytic and oxidative metabolism in GCs. This dual suppression of energy pathways could potentially influence ATP availability and may contribute to changes in follicular maturation and oocyte competence, which should be explored in future mechanistic studies.

This pilot, hypothesis-generating study provides essential preliminary data for the initial discovery of some metabolic alterations associated with PCOS; however, it has some limitations that should be recognized. First, the FF was collected at oocyte retrieval after controlled ovarian stimulation (COS) for ART, which may not accurately reflect the follicular environment under physiological conditions. Also, variations in the stimulation protocols and the dosages administered during COS could have influenced follicular metabolism. In addition, while the use of the HGrC1 granulosa cell line enabled a controlled and reproducible evaluation of metabolic patterns associated with FF exposure, this *in vitro* model cannot fully capture the complexity of the *in vivo* follicular niche. Thus, further mechanistic studies will be required to delineate the intracellular signaling pathways and regulatory networks underlying the observed metabolic phenotype, as well as to validate key findings in primary human granulosa cells.

The relatively small sample size represents another important limitation, as it restricts statistical power, particularly for formal testing of the interaction between PCOS and obesity. Accordingly, the study was not designed to test PCOS–obesity interactions in a factorial manner. Rather, its primary objective was to determine whether FF from women with PCOS, with or without concomitant obesity, was associated with detectable alterations in granulosa cell metabolism, thereby generating hypotheses for future, adequately powered mechanistic studies. We also acknowledge that certain functional and protein-level assessments of metabolic regulators were not performed, largely due to the limited volume of human FF available for experimental analyses, which constrained the number of mechanistic assays. To address these limitations, ongoing and future studies will aim to identify key FF components responsible for the observed effects and to establish mechanistic causality using low-input and highly sensitive approaches.

## 5 Conclusion

Collectively, these findings suggest that the coexistence of PCOS and obesity is associated with marked alterations in the metabolic interplay between the follicular environment and GCs. The combined downregulation of the transcripts *GLUT1* and glycolytic enzymes (*HK2, LDHA*), together with impaired glycolytic function, is consistent with a diminished capacity of GCs to metabolize glucose. The impaired glycolytic function was associated with an attenuated mitochondrial function, indicating a broader disruption in energy-related oxidative metabolic pathways. Taken together, the PCOS with obesity phenotype is characterized by alterations in FF composition that may compromise GCs bioenergetics, leading to energy insufficiency and potentially contributing to follicular arrest. These observations are preliminary and hypothesis-generating, highlighting the need for further studies to confirm these findings and clarify the underlying mechanisms.

## Data Availability

The names of the repository/repositories and accession number(s) can be found below: The datasets generated for this study can be found in the NIH Common Fund–supported National Metabolomics Data Repository (NMDR) through the Metabolomics Workbench platform. It has been assigned the Study ID ST004423 and is publicly accessible via the associated project DOI (10.21228/M8HC4V).

## References

[1] Joham AE Teede HJ Ranasinha S Zoungas S Boyle J . Prevalence of infertility and use of fertility treatment in women with polycystic ovary syndrome: data from a large community-based cohort study. J Women’s Health. (2015) 24:299–307. doi: 10.1089/jwh.2014.5000, PMID: 25654626

[2] Bozdag G Mumusoglu S Zengin D Karabulut E Yildiz BO . The prevalence and phenotypic features of polycystic ovary syndrome: a systematic review and meta-analysis. Hum Reprod. (2016) 31:2841–55. doi: 10.1093/humrep/dew218, PMID: 27664216

[3] Lizneva D Suturina L Walker W Brakta S Gavrilova-Jordan L Azziz R . Criteria, prevalence, and phenotypes of polycystic ovary syndrome. Fertil Steril. (2016) 106:6–15. doi: 10.1016/j.fertnstert.2016.05.003, PMID: 27233760

[4] Revised 2003 consensus on diagnostic criteria and long-term health risks related to polycystic ovary syndrome. Fertil Steril. (2004) 81:19–25. doi: 10.1093/humrep/deh098, PMID: 14711538

[5] Dapas M Dunaif A . Deconstructing a syndrome: genomic insights into PCOS causal mechanisms and classification. Endocr Rev. (2022) 43:927–65. doi: 10.1210/endrev/bnac001, PMID: 35026001 PMC9695127

[6] Siddiqui S Mateen S Ahmad R Moin S . A brief insight into the etiology, genetics, and immunology of polycystic ovarian syndrome (PCOS). J Assist Reprod Genet. (2022) 39:2439–73. doi: 10.1007/s10815-022-02625-7, PMID: 36190593 PMC9723082

[7] Singh S Pal N Shubham S Sarma DK Verma V Marotta F . Polycystic ovary syndrome: etiology, current management, and future therapeutics. J Clin Med. (2023) 12:1454. doi: 10.3390/jcm12041454, PMID: 36835989 PMC9964744

[8] Helvaci N Yildiz BO . Polycystic ovary syndrome as a metabolic disease. Nat Rev Endocrinol. (2025) 21:230–44. doi: 10.1038/s41574-024-01057-w, PMID: 39609634

[9] Stener-Victorin E Teede H Norman RJ Legro R Goodarzi MO Dokras A . Polycystic ovary syndrome. Nat Rev Dis Primers. (2024) 10:27. doi: 10.1038/s41572-024-00511-3, PMID: 38637590

[10] Broughton DE Moley KH . Obesity and female infertility: potential mediators of obesity’s impact. Fertil Steril. (2017) 107:840–7. doi: 10.1016/j.fertnstert.2017.01.017, PMID: 28292619

[11] Lim SS Norman RJ Davies MJ Moran LJ . The effect of obesity on polycystic ovary syndrome: a systematic review and meta-analysis. Obes Rev. (2013) 14:95–109. doi: 10.1111/j.1467-789X.2012.01053.x, PMID: 23114091

[12] Moran C Arriaga M Rodriguez G Moran S . Obesity differentially affects phenotypes of polycystic ovary syndrome. Int J Endocrinol 2012. (2012) 2012:317241. doi: 10.1155/2012/317241, PMID: 22829818 PMC3399368

[13] Vale-Fernandes E Moreira MV Bernardino RL Sousa D Brandão R Leal C . Polycystic ovary syndrome and excessive body weight impact independently and synergically on fertility treatment outcomes. Reprod Biol Endocrinol. (2025) 23:97. doi: 10.1186/s12958-025-01434-8, PMID: 40624700 PMC12232814

[14] Zhang L Feng Y Sun X Yi S Xiao X Ma F . Impact of body mass index on assisted reproductive technology outcomes in patients with polycystic ovary syndrome: a meta-analysis. Reprod BioMed Online. (2024) 48:103849. doi: 10.1016/j.rbmo.2024.103849, PMID: 38574459

[15] Rodgers RJ Irving-Rodgers HF . Formation of the ovarian follicular antrum and follicular fluid. Biol Reprod. (2010) 82:1021–9. doi: 10.1095/biolreprod.109.082941, PMID: 20164441

[16] Pan Y Pan C Zhang C . Unraveling the complexity of follicular fluid: insights into its composition, function, and clinical implications. J Ovarian Res. (2024) 17:237. doi: 10.1186/s13048-024-01551-9, PMID: 39593094 PMC11590415

[17] Dai M Hong L Yin T Liu S . Disturbed follicular microenvironment in polycystic ovary syndrome: relationship to oocyte quality and infertility. Endocrinology. (2024) 165. doi: 10.1210/endocr/bqae023, PMID: 38375912

[18] Przewocki J Łukaszuk A Jakiel G Wocławek-Potocka I Kłosińska K Olszewska J . Proteomic analysis of follicular fluid in polycystic ovary syndrome: insights into protein composition and metabolic pathway alterations. Int J Mol Sci. (2024) 25:11749. doi: 10.3390/ijms252111749, PMID: 39519300 PMC11546118

[19] Qian Y Tong Y Zeng Y Huang J Liu K Xie Y . Integrated lipid metabolomics and proteomics analysis reveal the pathogenesis of polycystic ovary syndrome. J Transl Med. (2024) 22:364. doi: 10.1186/s12967-024-05167-x, PMID: 38632610 PMC11022415

[20] Vale-Fernandes E Carrageta DF Moreira MV Guerra-Carvalho B Rodrigues B Sousa D . Follicular fluid profiling unveils anti-Müllerian hormone alongside glycolytic and mitochondrial dysfunction as markers of polycystic ovary syndrome. Mol Cell Endocrinol. (2025) 602:112536. doi: 10.1016/j.mce.2025.112536, PMID: 40185328

[21] Yu L Liu M Wang Z Liu T Liu S Wang B . Correlation between steroid levels in follicular fluid and hormone synthesis related substances in its exosomes and embryo quality in patients with polycystic ovary syndrome. Reprod Biol Endocrinol. (2021) 19:74. doi: 10.1186/s12958-021-00749-6, PMID: 34001150 PMC8127216

[22] Moreira MV Vale-Fernandes E Albergaria IC Alves MG Monteiro MP . Follicular fluid composition and reproductive outcomes of women with polycystic ovary syndrome undergoing *in vitro* fertilization: A systematic review. Rev Endocrine Metab Disord. (2023) 24:1045–73. doi: 10.1007/s11154-023-09819-z, PMID: 37493841 PMC10697886

[23] Vale-Fernandes E Moreira MV Rodrigues B Pereira SS Leal C Barreiro M . Anti-Müllerian hormone a surrogate of follicular fluid oxidative stress in polycystic ovary syndrome? Front Cell Dev Biol. (2024) 12:1408879. doi: 10.3389/fcell.2024.1408879, PMID: 39011395 PMC11246868

[24] Yan X Ma D Li R Xu J Zou Y He M . The role of mitochondrial dysfunction in ovarian granulosa cells in polycystic ovary syndrome. Endocr Connect. (2025) 14. doi: 10.1530/EC-25-0186, PMID: 40586348 PMC12268988

[25] Zhang Q Ren J Wang F Pan M Cui L Li M . Mitochondrial and glucose metabolic dysfunctions in granulosa cells induce impaired oocytes of polycystic ovary syndrome through Sirtuin 3. Free Radical Biol Med. (2022) 187:1–16. doi: 10.1016/j.freeradbiomed.2022.05.010, PMID: 35594990

[26] Zhao R Jiang Y Zhao S Zhao H . Multiomics analysis reveals molecular abnormalities in granulosa cells of women with polycystic ovary syndrome. Front Genet. (2021) 12:648701. doi: 10.3389/fgene.2021.648701, PMID: 34084179 PMC8168535

[27] Kotlyar AM Seifer DB . Women with PCOS who undergo IVF: a comprehensive review of therapeutic strategies for successful outcomes. Reprod Biol Endocrinol. (2023) 21:70. doi: 10.1186/s12958-023-01120-7, PMID: 37528417 PMC10391774

[28] Wishart DS Guo A Oler E Wang F Anjum A Peters H . HMDB 5.0: the human metabolome database for 2022. Nucleic Acids Res. (2022) 50:D622–d631. doi: 10.1093/nar/gkab1062, PMID: 34986597 PMC8728138

[29] Bayasula Iwase A Kiyono T Takikawa S Goto M Nakamura T . Establishment of a human nonluteinized granulosa cell line that transitions from the gonadotropin-independent to the gonadotropin-dependent status. Endocrinology. (2012) 153:2851–60. doi: 10.1210/en.2011-1810, PMID: 22467494

[30] Pfaffl MW . A new mathematical model for relative quantification in real-time RT-PCR. Nucleic Acids Res. (2001) 29:e45. doi: 10.1093/nar/29.9.e45, PMID: 11328886 PMC55695

[31] Bernardino RL Braga PC Oliveira PF Alves MG . Chapter 19 - Determination of glycolysis flux by extracellular flux measurements. In: Ferreira R Oliveira PF Nogueira-Ferreira R , editors. Glycolysis. Academic Press (2024). p. 443–54.

[32] Oliveira-Lopes B Braga PC Oliveira PF Alves MG Bernardino RL . A pharmacological dose of liraglutide improves mitochondrial performance in mouse leydig cells. Int J Mol Sci. (2025) 26:8903. doi: 10.3390/ijms26188903, PMID: 41009468 PMC12469514

[33] Freeman EW Sammel MD Lin H Gracia CR . Obesity and reproductive hormone levels in the transition to menopause. Menopause. (2010) 17:718–26. doi: 10.1097/gme.0b013e3181cec85d, PMID: 20216473 PMC2888623

[34] Stanikova D Luck T Pabst A Bae YJ Hinz A Glaesmer H . Associations between anxiety, body mass index, and sex hormones in women. Front Psychiatry. (2019) 10:479. doi: 10.3389/fpsyt.2019.00479, PMID: 31333520 PMC6620895

[35] Pratama G Wiweko B Asmarinah Widyahening IS Andraini T Bayuaji H . Mechanism of elevated LH/FSH ratio in lean PCOS revisited: a path analysis. Sci Rep. (2024) 14:8229. doi: 10.1038/s41598-024-58064-0, PMID: 38589425 PMC11002031

[36] Eng PC Phylactou M Qayum A Woods C Lee H Aziz S . Obesity-related hypogonadism in women. Endocr Rev. (2024) 45:171–89. doi: 10.1210/endrev/bnad027, PMID: 37559411 PMC10911953

[37] Jensterle M Weber M Pfeifer M Prezelj J Pfutzner A Janez A . Assessment of insulin resistance in young women with polycystic ovary syndrome. Int J Gynaecol Obstet. (2008) 102:137–40. doi: 10.1016/j.ijgo.2008.03.017, PMID: 18504045

[38] Muniyappa R Lee S Chen H Quon MJ . Current approaches for assessing insulin sensitivity and resistance *in vivo*: advantages, limitations, and appropriate usage. Am J Physiol Endocrinol Metab. (2008) 294:E15–26. doi: 10.1152/ajpendo.00645.2007, PMID: 17957034

[39] Tsai S-F Yang C-T Liu W-J Lee C-L . Development and validation of an insulin resistance model for a population without diabetes mellitus and its clinical implication: a prospective cohort study. eClinicalMedicine. (2023) 58:101934. doi: 10.1016/j.eclinm.2023.101934, PMID: 37090441 PMC10119497

[40] Wongwananuruk T Rattanachaiyanont M Leerasiri P Indhavivadhana S Techatraisak K Angsuwathana S . The usefulness of homeostatic measurement assessment-insulin resistance (HOMA-IR) for detection of glucose intolerance in thai women of reproductive age with polycystic ovary syndrome. Int J Endocrinol 2012. (2012) p:571035. doi: 10.1155/2012/571035, PMID: 22737168 PMC3378956

[41] Eppig J . Oocyte control of ovarian follicular development and function in mammals. Reproduction. (2001) 122:829–38. doi: 10.1530/rep.0.1220829, PMID: 11732978

[42] Sugiura K Pendola FL Eppig JJ . Oocyte control of metabolic cooperativity between oocytes and companion granulosa cells: energy metabolism. Dev Biol. (2005) 279:20–30. doi: 10.1016/j.ydbio.2004.11.027, PMID: 15708555

[43] Sutton-McDowall ML Gilchrist RB Thompson JG . The pivotal role of glucose metabolism in determining oocyte developmental competence. Reproduction. (2010) 139:685–95. doi: 10.1530/REP-09-0345, PMID: 20089664

[44] Xie H-L Wang Y-B Jiao G-Z Kong D-L Li Q Li H . Effects of glucose metabolism during *in vitro* maturation on cytoplasmic maturation of mouse oocytes. Sci Rep. (2016) 6:20764. doi: 10.1038/srep20764, PMID: 26857840 PMC4746733

[45] Zhang X Zhang W Wang Z Zheng N Yuan F Li B . Enhanced glycolysis in granulosa cells promotes the activation of primordial follicles through mTOR signaling. Cell Death Dis. (2022) 13:87. doi: 10.1038/s41419-022-04541-1, PMID: 35087042 PMC8795455

[46] Li S Wu J Lu R Zhou B Dai H Zhang Z . Mogroside V restores glycolytic function via LDHA promoter demethylation independent of alternative sp*licing in PCOS granulosa cells*. J Steroid Biochem Mol Biol. (2025) 254:106839. doi: 10.1016/j.jsbmb.2025.106839, PMID: 40738262

[47] Zhao B Fan L Liu M Wu H Zhang Y Shen Q . Androgen-induced lactic acid accumulation contributes to the apoptosis of ovarian granulosa cells in polycystic ovary syndrome mice. Antioxid (Basel). (2025) 14:1235. doi: 10.3390/antiox14101235, PMID: 41154544 PMC12561039

[48] Zhu R Yang L Li Y . TXNIP participates in the pathogenesis of PCOS by regulating glycolysis in granulosa cells. Biochem Biophys Res Commun. (2025) 775:152149. doi: 10.1016/j.bbrc.2025.152149, PMID: 40499496

[49] Mu L Ye Z Hu J Zhang Y Chen K Sun H . PPM1K-regulated impaired catabolism of branched-chain amino acids orchestrates polycystic ovary syndrome. EBioMedicine. (2023) 89:104492. doi: 10.1016/j.ebiom.2023.104492, PMID: 36863088 PMC9986518

[50] Cao J Huo P Cui K Wei H Cao J Wang J . Follicular fluid-derived exosomal miR-143-3p/miR-155-5p regulate follicular dysplasia by modulating glycolysis in granulosa cells in polycystic ovary syndrome. Cell Commun Signal. (2022) 20:61. doi: 10.1186/s12964-022-00876-6, PMID: 35534864 PMC9082924

[51] Cui X Lei X Huang T Mao X Shen Z Yang X . Follicular fluid-derived extracellular vesicles miR-34a-5p regulates granulosa cell glycolysis in polycystic ovary syndrome by targeting LDHA. J Ovarian Res. (2024) 17:223. doi: 10.1186/s13048-024-01542-w, PMID: 39538292 PMC11562512

[52] Tang Q Tan P Ma N Ma X . Physiological functions of threonine in animals: beyond nutrition metabolism. Nutrients. (2021) 13:2592. doi: 10.3390/nu13082592, PMID: 34444752 PMC8399342

[53] Castiglione Morelli MA Iuliano A Schettini SCA Petruzzi D Ferri A Colucci P . NMR metabolic profiling of follicular fluid for investigating the different causes of female infertility: a pilot study. Metabolomics. (2019) 15:19. doi: 10.1007/s11306-019-1481-x, PMID: 30830455

[54] Lazzarino G Pallisco R Bilotta G Listorti I Mangione R Saab MW . Altered follicular fluid metabolic pattern correlates with female infertility and outcome measures of *in vitro* fertilization. Int J Mol Sci. (2021) 22:8735. doi: 10.3390/ijms22168735, PMID: 34445441 PMC8395780

[55] Siemers KM Klein AK Baack ML . Mitochondrial dysfunction in PCOS: insights into reproductive organ pathophysiology. Int J Mol Sci. (2023) 24(17):13123. doi: 10.3390/ijms241713123, PMID: 37685928 PMC10488260

[56] Sreerangaraja Urs DB Wu WH Komrskova K Postlerova P Lin YF Tzeng CR . Mitochondrial function in modulating human granulosa cell steroidogenesis and female fertility. Int J Mol Sci. (2020) 21. doi: 10.3390/ijms21103592, PMID: 32438750 PMC7279321

[57] Yan H Wang L Zhang G Li N Zhao Y Liu J . Oxidative stress and energy metabolism abnormalities in polycystic ovary syndrome: from mechanisms to therapeutic strategies. Reprod Biol Endocrinol. (2024) 22:159. doi: 10.1186/s12958-024-01337-0, PMID: 39722030 PMC11670460

[58] Zhao YK Gao YN Wang LC Wang J Wang GJ Wu HL . Correlation between abnormal energy metabolism of ovarian granulosa cells and *in vitro* fertilization-embryo transfer outcomes in patients with polycystic ovary syndrome and obesity. J Ovarian Res. (2023) 16:145. doi: 10.1186/s13048-023-01204-3, PMID: 37480140 PMC10362761

[59] Huang L Qin X Tian C Ling S Luo X Huang B . Association between mitochondrial SIRTs (SIRT3, SIRT4, and SIRT5) and PCOS. Eur J Med Res. (2025) 30:611. doi: 10.1186/s40001-025-02862-3, PMID: 40640941 PMC12243299

[60] Pang X Cheng J Wu T Sun L . SIRT3 ameliorates polycystic ovary syndrome through FOXO1/PGC-1α signaling pathway. Endocrine. (2023) 80:201–11. doi: 10.1007/s12020-022-03262-x, PMID: 36598711

[61] Wild RA Rizzo M Clifton S Carmina E . Lipid levels in polycystic ovary syndrome: systematic review and meta-analysis. Fertil Steril. (2011) 95:1073–9.e1–11. doi: 10.1016/j.fertnstert.2010.12.027, PMID: 21247558

[62] Liu L Yin TL Chen Y Li Y Yin L Ding J . Follicular dynamics of glycerophospholipid and sp*hingolipid metabolisms in polycystic ovary syndrome patients*. J Steroid Biochem Mol Biol. (2019) 185:142–9. doi: 10.1016/j.jsbmb.2018.08.008, PMID: 30121347

[63] Niu Z Lin N Gu R Sun Y Feng Y . Associations between insulin resistance, free fatty acids, and oocyte quality in polycystic ovary syndrome during *in vitro* fertilization. J Clin Endocrinol Metab. (2014) 99:E2269–76. doi: 10.1210/jc.2013-3942, PMID: 24694334 PMC4223443

[64] Sun Z Chang HM Wang A Song J Zhang X Guo J . Identification of potential metabolic biomarkers of polycystic ovary syndrome in follicular fluid by SWATH mass sp*ectrometry*. Reprod Biol Endocrinol. (2019) 17:45. doi: 10.1186/s12958-019-0490-y, PMID: 31186025 PMC6560878

[65] Lai Y Ye Z Mu L Zhang Y Long X Zhang C . Elevated levels of follicular fatty acids induce ovarian inflammation via ERK1/2 and inflammasome activation in PCOS. J Clin Endocrinol Metab. (2022) 107:2307–17. doi: 10.1210/clinem/dgac281, PMID: 35521772

[66] Ma Y Zheng L Wang Y Gao Y Xu Y . Arachidonic acid in follicular fluid of PCOS induces oxidative stress in a human ovarian granulosa tumor cell line (KGN) and upregulates GDF15 expression as a response. Front Endocrinol (Lausanne). (2022) 13:865748. doi: 10.3389/fendo.2022.865748, PMID: 35634503 PMC9132262

